# Two new species and three new records of Cuprininae (Lepidoptera, Stathmopodidae) from Jeju Island, Korea, with molecular evidence for their placement

**DOI:** 10.3897/zookeys.1287.194473

**Published:** 2026-08-04

**Authors:** Ga-Eun Lee, Takeshi Terada

**Affiliations:** 1 The Research Institute for Basic Sciences, Jeju National University, Jeju 63243, Republic of Korea The Research Institute for Basic Sciences, Jeju National University Jeju Republic of Korea; 2 Okayama Prefectural Environmental Conservation Foundation, Inc., Okayama, Japan Okayama Prefectural Environmental Conservation Foundation, Inc. Okayama Japan

**Keywords:** *
Calicotis
*, *
Cuprina
*, fern-feeding moth, molecular data, *
Pediformis
*, *
Petala
*, taxonomy

## Abstract

Cuprininae is a distinctive subfamily within Stathmopodidae whose larvae feed exclusively on fern spores. The group currently comprises 81 described species distributed throughout the Old World; however, the Korean fauna currently includes only three species in two genera, *Calicotis* Meyrick, 1889 and *Cuprina* Sinev, 1988. In this study, two new species *Pediformis astathmeta***sp. nov**. and *Calicotis atricosta***sp. nov**. are described, and three species *Cuprina porphyrantha* (Meyrick, 1913), *Petala aedificatrix* (Terada, 2016) and *P. margaritacea* (Terada, 2016) are reported from Korea for the first time. All material examined was collected from Jeju Island, the southernmost and largest island of Korea. To verify the generic placement and species-level identification of these taxa, phylogenetic analyses were conducted using five gene regions (COI, EF-1α, CAD, H3, and Wg). Both maximum likelihood and Bayesian inference analyses yielded largely congruent topologies and the species examined were consistently placed within their respective genera and grouped with conspecific sequences. A distribution map, photographs of adults and genitalia, and DNA sequence information are provided.

## Introduction

Cuprininae represents a small but distinctive lineage within the family Stathmopodidae, notable for its specialized ecological association with ferns. Larvae of this subfamily exclusively utilize fern spores, a feeding habit considered to be a rare trait not only in Lepidoptera but also in insects generally (Sawamura et al. 2009; Fuentes-Jacques et al. 2022a, 2022b; Shen et al. 2024). According to a recent evolutionary study on fern-spore-feeding Stathmopodidae, this trait likely evolved only once within the family and has been reported from Cuprininae and two other genera, *Agrioscelis* and *Erineda* (Shen et al. 2024). The same study also suggested that oligophagy was the ancestral host-use state in Cuprininae rather than specialization on a single fern genus or family. To date, at least 11 fern families, including Aspleniaceae, Athyriaceae, Dennstaedtiaceae, Dryopteridaceae, Nephrolepidaceae, Onocleaceae, Plagiogyriaceae, Polypodiaceae, Pteridaceae, Thelypteridaceae and Tectariaceae, have been recorded as host plants of Cuprininae (Terada 2016; Shen et al. 2024).

Morphologically, Cuprininae is characterized by its small body size, a dorsoventrally flattened head, and male antennae with or without short ciliae (Shen et al. 2024). The subfamily was originally established to include seven genera by Sinev (2015): *Actinoscelis* Meyrick, 1912, *Calicotis* Meyrick, 1889, *Cuprina* Sinev, 1988, *Lissocnemitis* Meyrick, 1934, *Pachyrhabda* Meyrick, 1897, *Thylacosceles* Meyrick, 1889 and *Thylacosceloides* Sinev, 1988. More recently, Shen et al. (2024) conducted a comprehensive phylogenetic revision based on one mitochondrial and four nuclear genes, resulting in substantial changes to the generic classification. In their study, three new genera (*Trigonoda*, *Petala* and *Pediformis*) were proposed whereas *Lissocnemitis* and *Pachyrhabda* were synonymized with *Calicotis*, leading to the recognition of eight genera within Cuprininae.

Cuprininae is distributed in the Eastern Palearctic, the Oriental, the Oceanian, the Australasian, and the Afrotropical regions and currently comprises 81 described species (Shen et al. 2024). In Korea, knowledge of the subfamily remains limited, with only three species, *Calicotis chrysoptera* Terada, 2016, *C. latebrifica* Terada, 2016 and *Cuprina fuscella* Sinev, 1988 reliably recorded to date (Koo et al. 2018; Sohn 2023; Jeong and Kim 2025). Although *Pachyrhabda benearena* Jeong & Kim, 2024 was recently described from Korea (Jeong and Kim 2024), its taxonomic placement requires further examination and is therefore not treated here as a confirmed member of Cuprininae.

In the present study, we describe two new species belonging to *Calicotis* and *Pediformis* and report three additional species from the genera *Cuprina* and *Petala* as new records for Korea. All examined specimens were collected from Jeju Island (also known as Jeju-do or Jeju Special Self-Governing Province), the largest volcanic island located at the southernmost part of the Korean Peninsula. In addition to detailed morphological examinations, we provide DNA sequence data and phylogenetic analyses to support generic placement and species delimitation, and to briefly discuss phylogenetic relationships within Cuprininae in the context of recent molecular studies.

Here, we present diagnoses, photographs of adults and genitalia, distributional information, and molecular data for all species treated in this paper.

### Abbreviations

**JNU** Specimen Room of Bioresources, Jeju National University, Jeju City, Korea;

**NHMUK** Natural History Museum, Department of Zoology, London, United Kingdom;

**NIBR** National Institute of Biological Resources, Incheon, Korea;

**OMU** Osaka Metropolitan University, Osaka Prefecture, Japan;

**TD** Type depository;

**TL** Type locality.

## Materials and methods

### Taxon sampling and morphological methods

We morphologically examined seven species of Cuprininae, including two new species, three newly recorded species, and two previously recorded species (*Calicotis latebrifica* and *Cuprina fuscella*) from Korea. All materials were collected from Jeju Island in Korea between 2020 and 2025 using light traps equipped with 300–400 W mercury vapor or metal-halide lamps, as well as insect nets during daytime surveys. Host plants were investigated at the collecting sites, and larvae of four cuprinine species were found feeding on five fern species in Dryopteridaceae: *Arachniodes aristata* (G.Forst.) Tindale, *A. sporadosora* (Kunze) Nakaike, *Cyrtomium falcatum* var. *devexiscapulae* (Koidz.) Tagawa, *Dryopteris uniformis* (Makino) Makino and *Polystichum polyblepharum* (Roem. ex Kunze) C.Presl. Collected larvae were reared indoors and subsequently included in molecular analyses.

Genitalia and wing slides were prepared by maceration in 10% KOH, followed by cleaning, staining with Eosin Y and Chlorazol Black E, and mounting in Euparal or Canada balsam. Photographs were taken using a Dhyana 400DC camera (Tucsen Photonics, China) attached to a Leica Z16 APO stereomicroscope (Leica Microsystems, Wetzlar, Germany), and images were finally processed using Adobe Photoshop® CS6. A distribution map was generated using DIVA-GIS 7.5 (Hijmans et al. 2012). Adult specimens and genitalia slides are deposited in JNU and NIBR; detailed depository information for each specimen is provided in the taxonomic section.

Morphological terminology generally follows Terada (2016), except that the terms ‘antrum’ (Kristensen 2003) and ‘apical spatulate projection’ (Koster and Sinev 2003) are used. The term ‘antrum’ refers to the cup-shaped part situated posterior to the ductus bursae in the female genitalia, and ‘apical spatulate projection’ refers to the spatulate structure located at the apex of the aedeagus.

### Molecular data and phylogenetic analyses

For the molecular study, genomic DNA was extracted from one or two legs of dried adult specimens representing seven species and from larvae of four species. We amplified one mitochondrial protein-coding gene, cytochrome *c* oxidase subunit I (COI) and four nuclear genes, EF-1α, carbamoyl-phosphate synthase domain (CAD), histone H3 (H3), and wingless (Wg), following the phylogenetic framework of Shen et al. (2024). DNA extraction followed Lee et al. (2021), and PCR primers were used as described in Shen et al. (2024). The sequences were edited and aligned manually in MEGA12 (Kumar et al. 2024) and registered in GenBank (Table 1). All extracted genomic DNA is deposited in JNU.

**Table 1.** Specimen vouchers and GenBank accession numbers for species included in this study.

**Species name**	**Specimen voucher no**.	**GenBank accession no**.				
		**COI**	**EF–1α**	**CAD**	**H3**	**Wg**
*Pediformis astathmeta* sp. nov.	LGE-G550	PZ209901	–	–	–	–
	LGE-G895	PZ209902	–	–	–	–
	LGE-G988	PZ209903	–	–	–	–
	LGE-G1020	PZ209904	–	–	–	–
	LGE-G1022	PZ209905	–	–	–	–
	LGE-G1039	PZ209906	PZ231427	–	–	–
	LGE-G1040	PZ209907	PZ231428	–	–	–
	LGE-G1062	PZ209908	–	PZ231430	PZ231431	PZ231434
*Calicotis atricosta* sp. nov.	LGE-G549	PZ209909	PZ231429	–	PZ231432	–
	LGE-G901	PZ209910	–	–	PZ231433	–
*C. latebrifica*	LGE-G552	PZ209911	–	–	–	–
	LGE-G899	PZ209912	–	–	–	–
	LGE-G900	PZ209913	–	–	–	–
	LGE-G1006	PZ209914	–	–	–	–
	LGE-G1015	PZ209915	–	–	–	–
	LGE-G1038	PZ209916	–	–	–	–
*Cuprina fuscella*	LGE-G738	PZ209917	–	–	–	–
	LGE-G1000	PZ209918	–	–	–	–
	LGE-G1001	PZ209919	–	–	–	–
	LGE-G1005	PZ209920	–	–	–	–
	LGE-G1014	PZ209921	–	–	–	–
	LGE-G1016	PZ209922	–	–	–	–
	LGE-G1017	PZ209923	–	–	–	–
	LGE-G1018	PZ209924	–	–	–	–
	LGE-G1019	PZ209925	–	–	–	–
	LGE-G1021	PZ209926	–	–	–	–
*C. porphyrantha*	LGE-G548	PZ209927	–	–	–	–
	LGE-G1023	PZ209928	–	–	–	–
*Petala aedificatrix*	LGE-G897	PZ209929	–	–	–	–
*P. margaritacea*	LGE-G551	PZ209930	–	–	–	–
	LGE-G894	PZ209931	–	–	–	–
	LGE-G896	PZ209932	–	–	–	–
	LGE-G898	PZ209933	–	–	–	–

To reconstruct phylogenetic relationships and to verify both the generic placement of new species and the species-level identification of the newly recorded taxa, we additionally included DNA sequences representing 37 cuprinine species and one outgroup taxon, *Stathmopoda auriferella* (Walker, 1864) (Stathmopodinae), obtained from GenBank and BOLD. The five genes were concatenated into a single alignment with a total length of 2,578 bp. Sequences were concatenated on a specimen basis, and each terminal represented a single specimen. Missing loci were coded as gaps, and no composite species-level terminals were constructed from multiple individuals.

Maximum likelihood (ML) and Bayesian inference (BI) analyses were conducted on the CIPRES Science Gateway (Miller et al. 2010) using IQ-TREE v3.0.1 (Wong et al. 2025) and MrBayes v3.2.7 (Ronquist et al. 2012), respectively. The concatenated dataset was initially partitioned by gene and codon position (15 partitions). For the ML analysis, ModelFinder was employed to select the best-fit partitioning scheme and substitution models under the Bayesian Information Criterion (BIC). This procedure resulted in a merged partition scheme consisting of nine partitions for the final ML analysis. Branch support was assessed using 1,000 ultrafast bootstrap (UFBoot) replicates and 1,000 SH-like approximate likelihood ratio test (SH-aLRT) replicates, with the -bnni option applied to reduce potential overestimation of support values, following Shen et al. (2024).

Bayesian inference was performed using the GTR + Γ substitution model for all 15 codon partitions. Although the same substitution model was applied across partitions, model parameters were estimated independently for each partition by unlinking them in MrBayes. Two independent runs of four Markov chains were executed for 10 million generations, sampling every 1,000 generations. Convergence was assessed by examining the average standard deviation of split frequencies between runs, which decreased below 0.01. The first 25% of samples were discarded as burn-in, and a 50% majority-rule consensus tree was generated to obtain posterior probability (PP) values. UFBoot values ≥ 95 and posterior probabilities ≥ 0.95 were considered strong support.

## Taxonomic account

### Family Stathmopodidae Meyrick, 1913

**Subfamily Cuprininae Sinev, 2015**

#### Genus *Pediformis* Shen & Hsu, 2024

##### Pediformis astathmeta sp. nov.

Taxon classificationAnimaliaLepidopteraStathmopodidae

5D6FF8BA-8C58-5444-9795-B43D8282D1EC

https://zoobank.org/0CEAB4A7-BC95-41F0-9CC4-64D5CB17D97D

**Figure 1.**
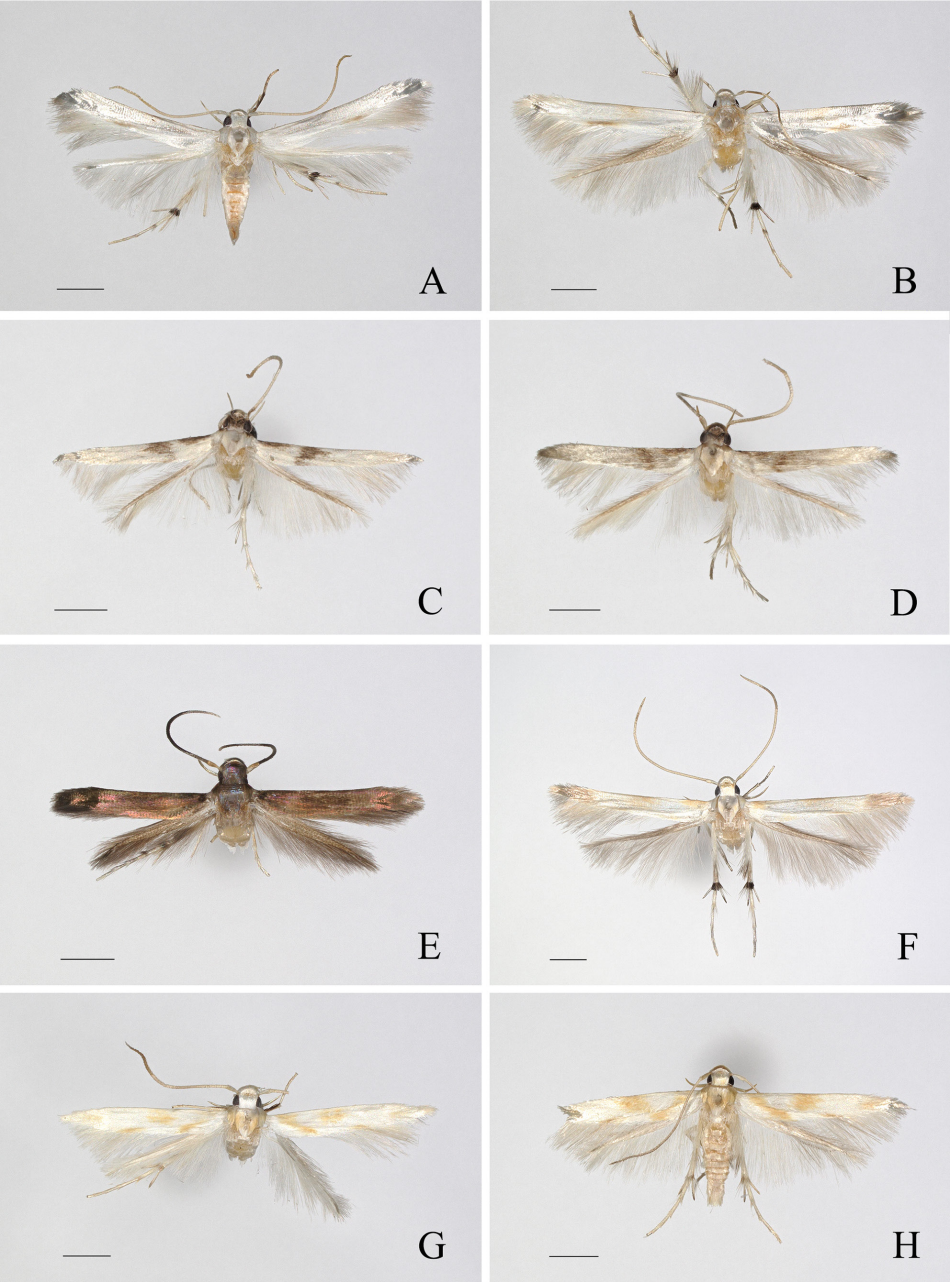
Adults of Cuprininae species. **A**. *Pediformis astathmeta* sp. nov., holotype, male; **B**. Ditto, paratype, female; **C**. *Calicotis atricosta* sp. nov., holotype, male; **D**. *Calicotis atricosta* sp. nov., paratype, male; **E**. *Cuprina porphyrantha* (Meyrick, 1913), male; **F**. *Petala aedificatrix* (Terada, 2016), male; **G**. *P. margaritacea* (Terada, 2016), male; **H**. Ditto, female. Scale bars: 1.0 mm.

**Figure 2.**
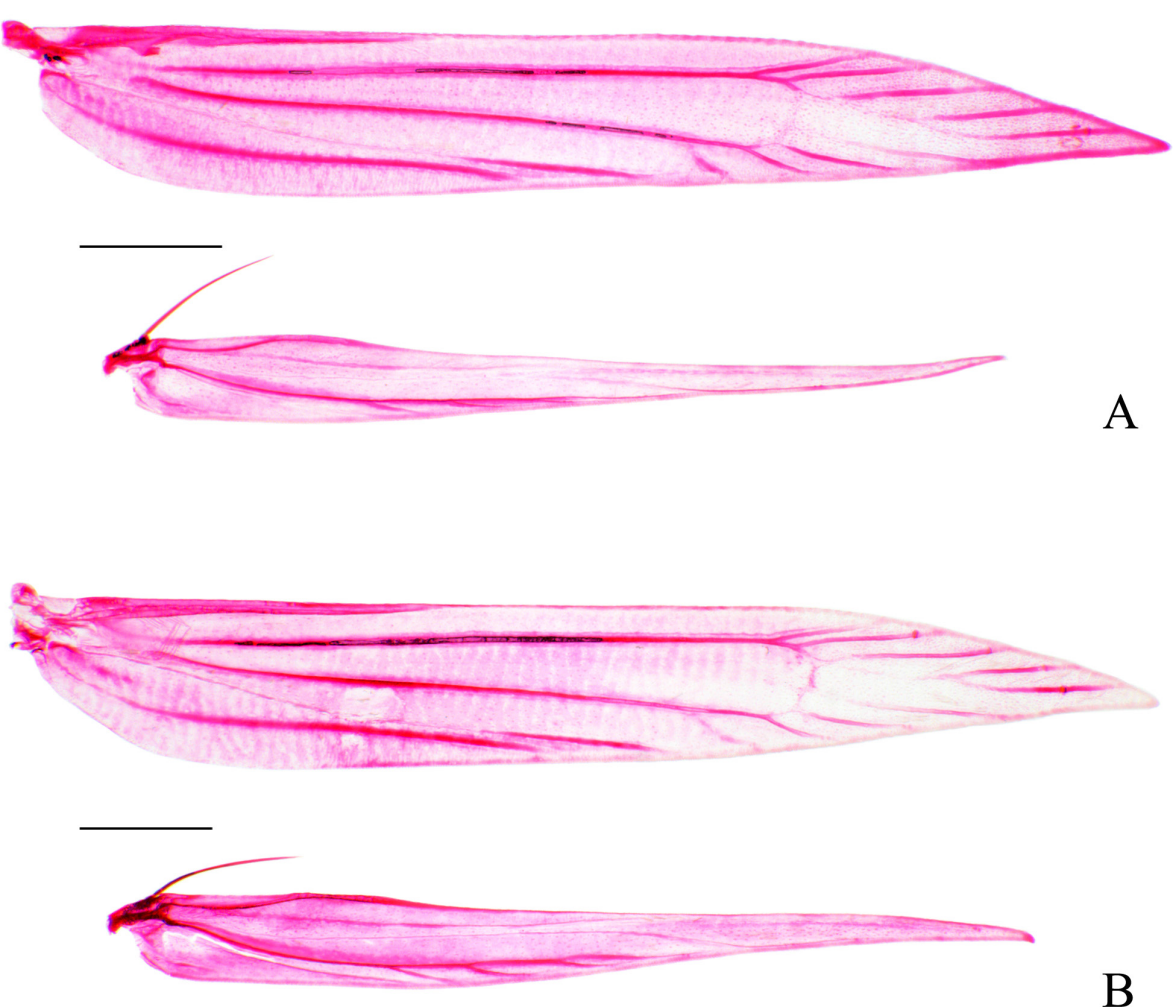
Wing venations of *Pediformis astathmeta* sp. nov. **A**. Paratype, male, slide no. LGE25043W; **B**. Paratype, female, slide no. LGE25044W. Scale bars: 0.5 mm.

**Figure 3.**
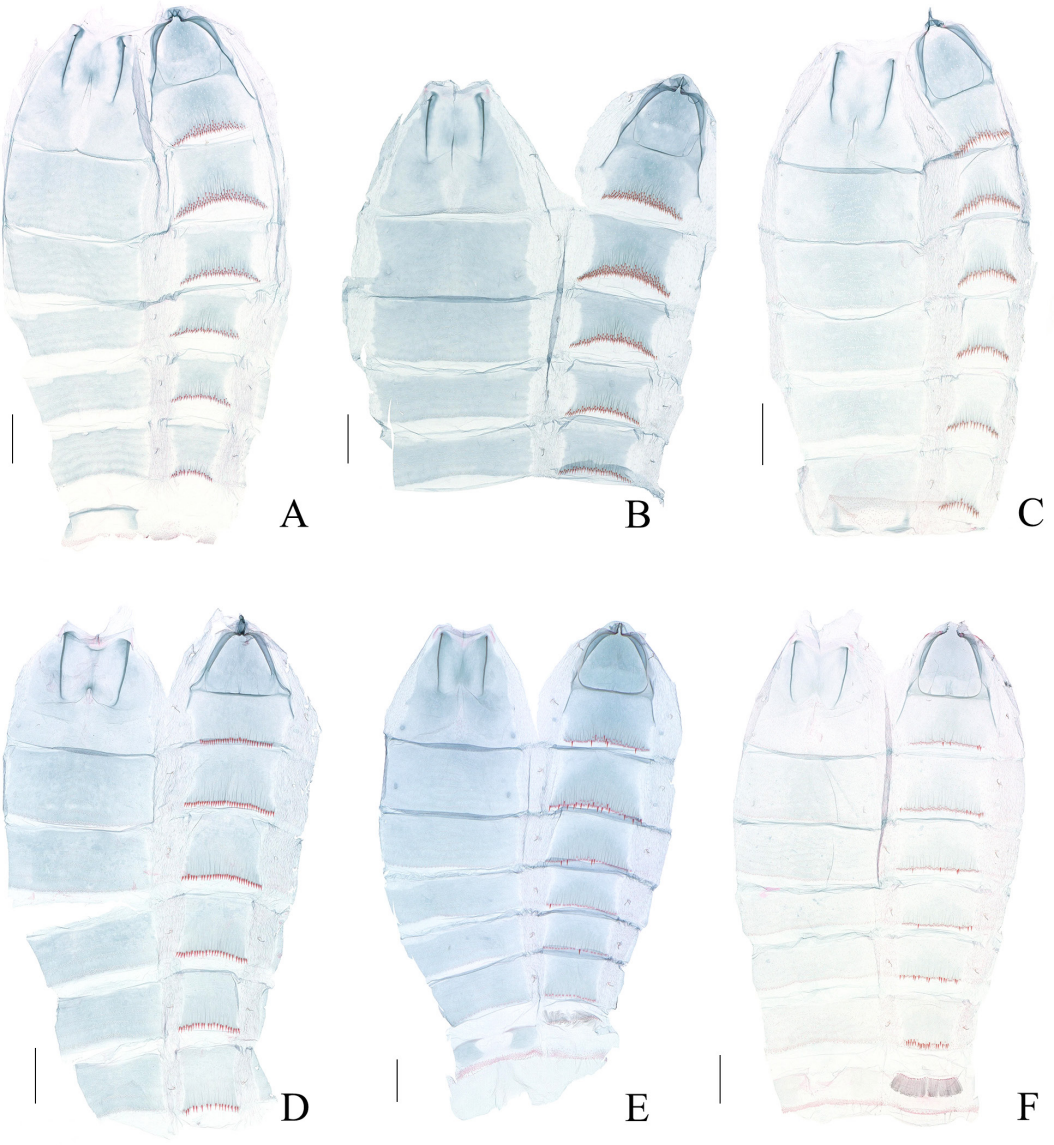
Abdomens of Cuprininae species. **A**. *Pediformis astathmeta* sp. nov., paratype, male, slide no. LGE22021; **B**. Ditto, paratype, female, slide no. LGE25015; **C**. *Calicotis atricosta* sp. nov., holotype, male, slide no. LGE22020; **D**. *Cuprina porphyrantha* (Meyrick, 1913), male, slide no. LGE22033; **E**. *Petala aedificatrix* (Terada, 2016), male, slide no. LGE24019; **F**. *P. margaritacea* (Terada, 2016), male, slide no. LGE20014. Scale bars: 0.25 mm.

**Figure 4.**
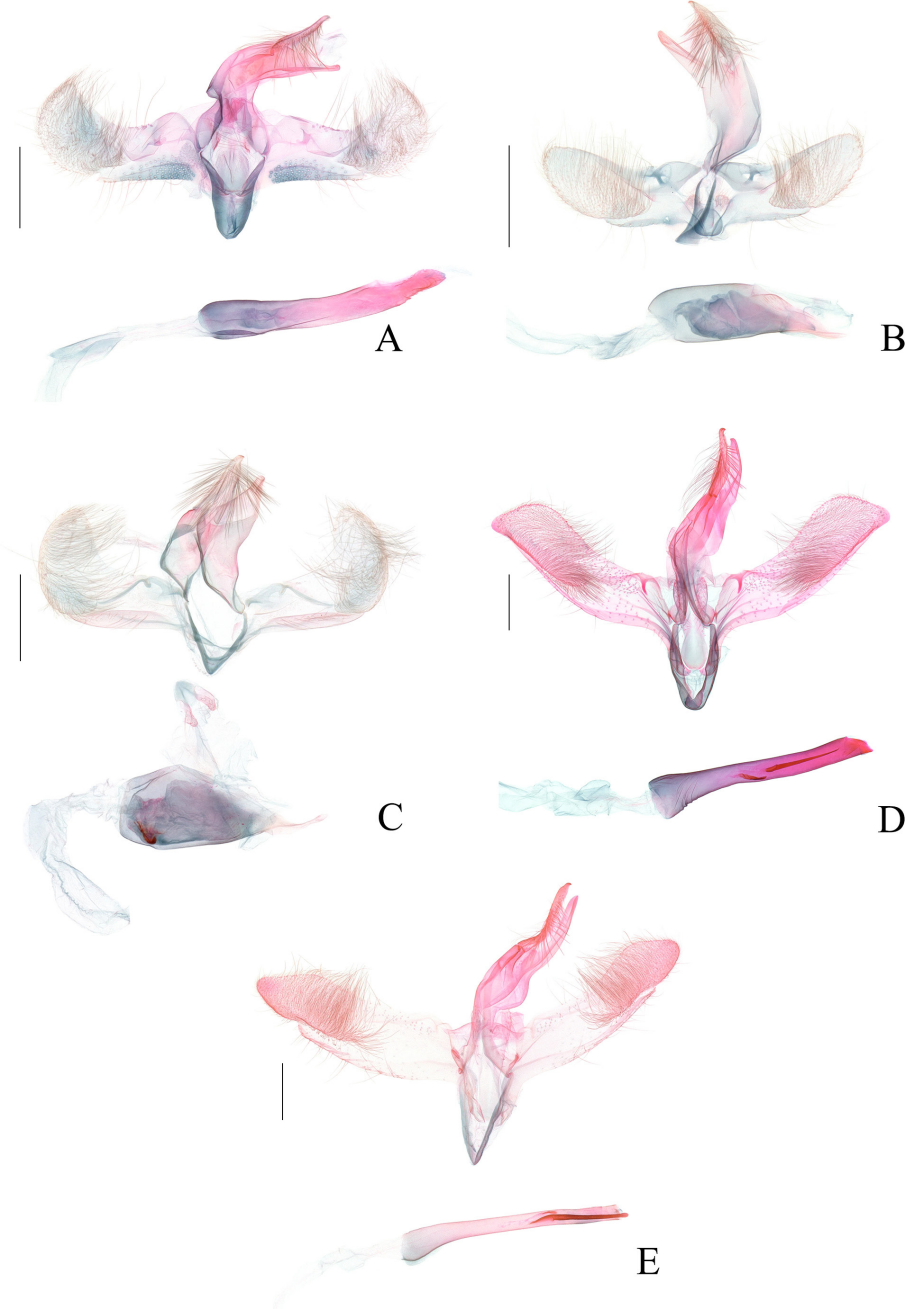
Male genitalia of Cuprininae species. **A**. *Pediformis astathmeta* sp. nov., paratype, slide no. LGE22021; **B**. *Calicotis atricosta* sp. nov., holotype, slide no. LGE22020; **C**. *Cuprina porphyrantha* (Meyrick, 1913), slide no. LGE22033; **D**. *Petala aedificatrix* (Terada, 2016), slide no. LGE24019; **E**. *P. margaritacea* (Terada, 2016), slide no. LGE20014. Scale bars: 0.2 mm.

**Figure 5.**
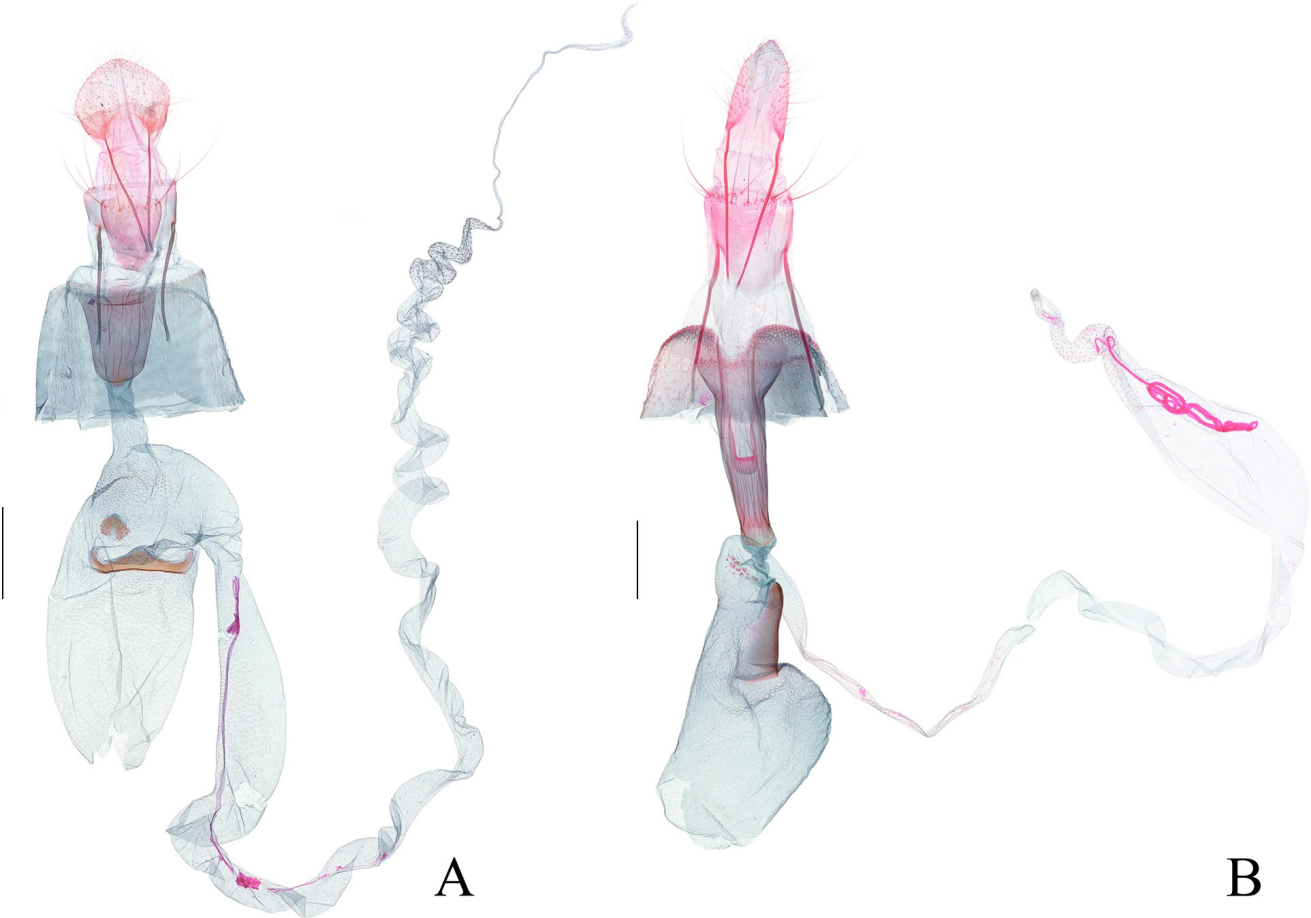
Female genitalia of Cuprininae species. **A**. *Pediformis astathmeta* sp. nov., paratype, slide no. LGE25015; **B**. *Petala margaritacea* (Terada, 2016), slide no. LGE24020. Scale bars: 0.25 mm.

**Figure 6.**
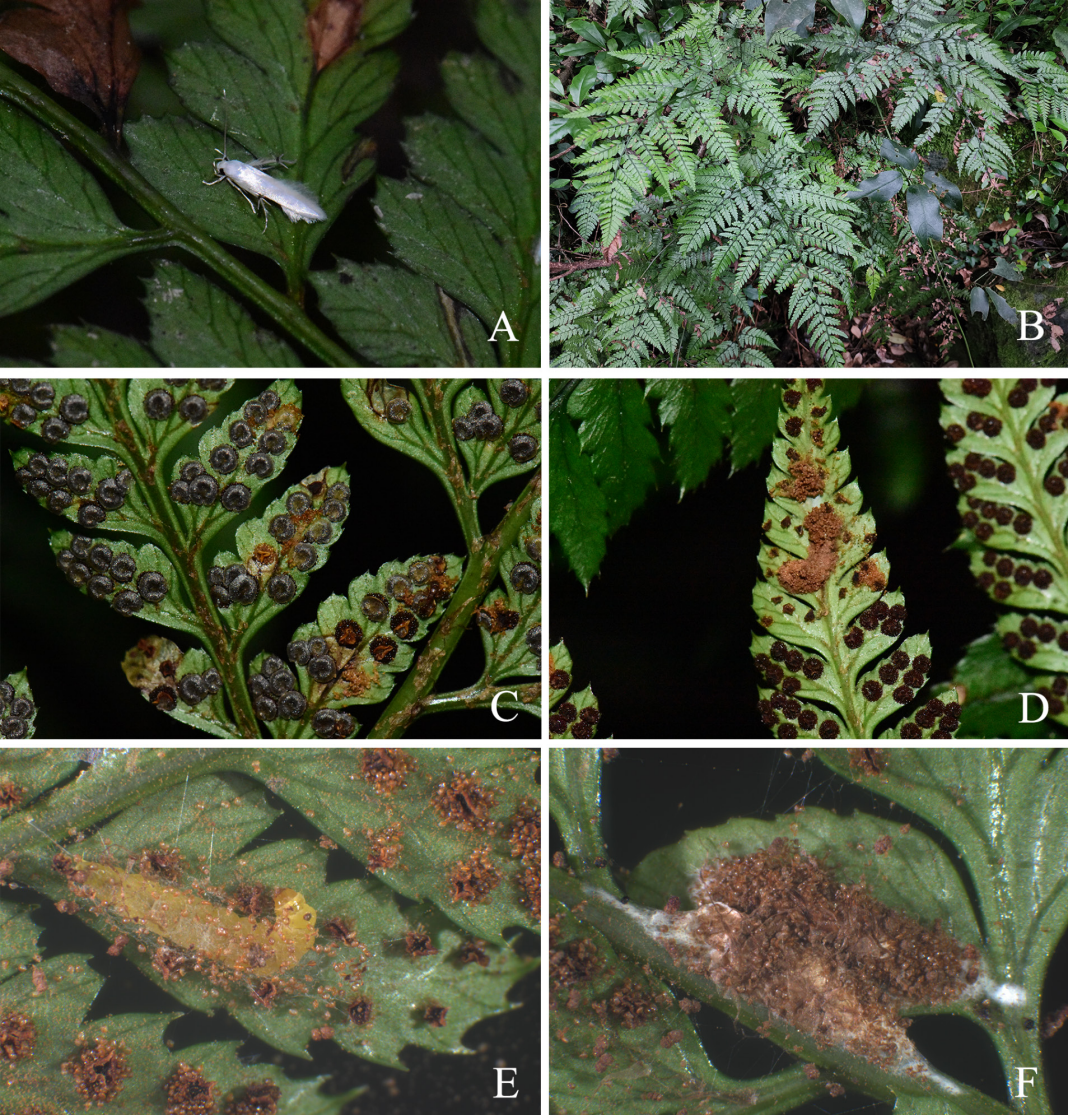
*Pediformis astathmeta* sp. nov. in its habitat. **A**. Adult; **B**. Host plant, *Arachniodes sporadosora* (Kunze) Nakaike; **C**. Fern fronds infested by early instar larvae; **D**. Tunnel-like shelters on the underside of fern fronds; **E**. Late instar larva forming a silky gallery; **F**. Cocoons.

**Figure 7.**
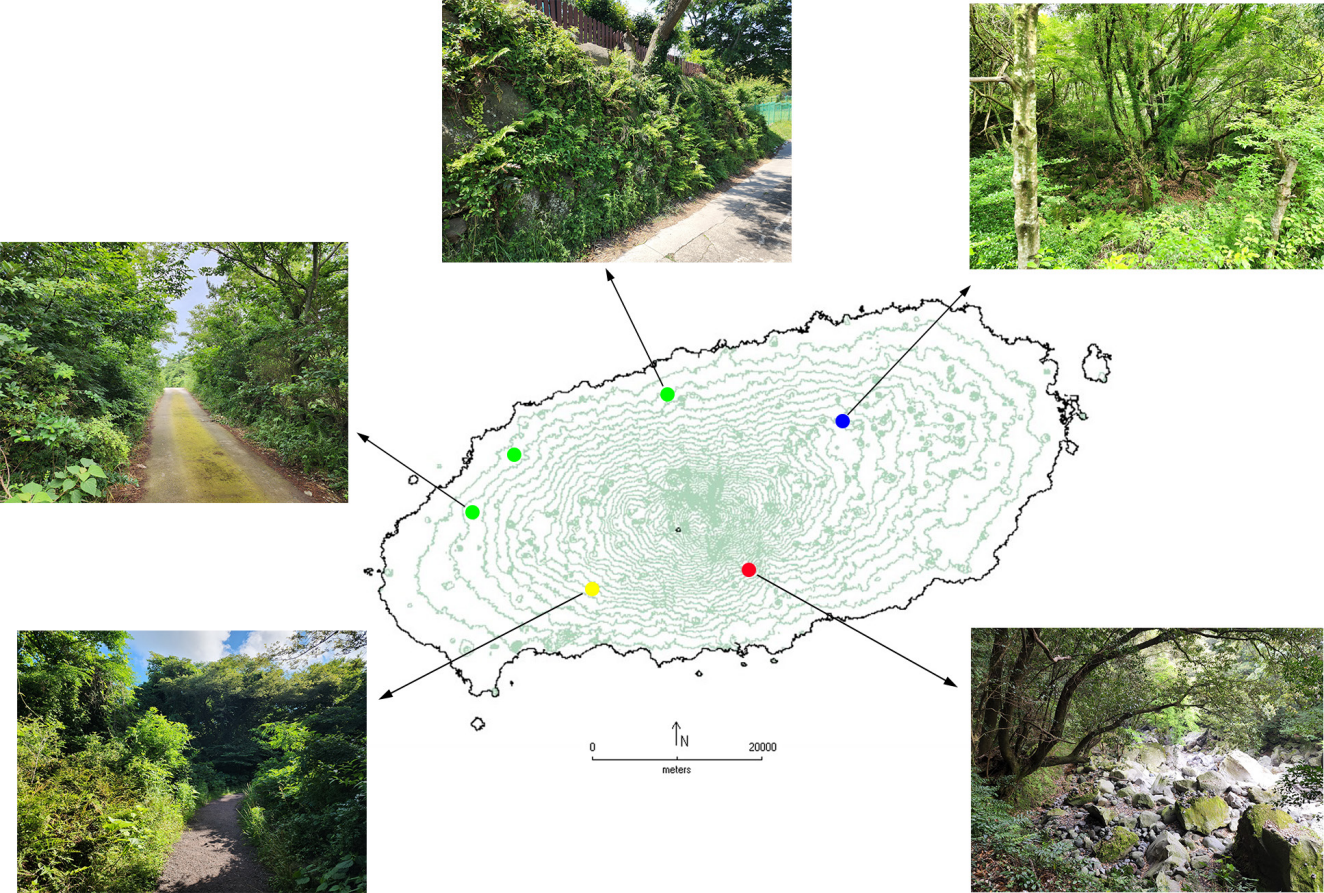
Distribution and habitats of Cuprininae species on Jeju Island, Korea. A red circle indicates a collection site where *Pediformis astathmeta* sp. nov., *Calicotis atricosta* sp. nov., and *Cuprina porphyrantha* (Meyrick, 1913) were collected together; a blue circle indicates a collection site where *Petala aedificatrix* (Terada, 2016) was collected; a yellow circle indicates a collection site where only *Cuprina porphyrantha* (Meyrick, 1913) was collected; green circles indicate collection sites where *P. margaritacea* (Terada, 2016) was collected.

Figs 1A, 1B, 2, 3A, 3B, 4A, 5A, 6, 7

###### Korean name.

Je-ju-go-sa-ri-kkok-ji-na-bang.

###### Type material.

***Holotype***: Korea • ♂; Jeju-do, Seogwipo-si, Topyeong-dong; 23.V.2021; Ga-Eun Lee leg.; genitalia slide no. LGE25031 (JNU); DNA voucher no. LGE-G988. ***Paratypes***: Korea • 12♂11♀, same original data as holotype; genitalia slide nos LGE22021m, LGE22022f, LGE25014m, LGE25015f, LGE25016f, LGE25031m, LGE25042m (all deposited in JNU except for one female in NIBR); wing slide nos LGE25042W, LGE25043W, LGE25044W; DNA voucher nos LGE-G550, -G895 • 3♂1♀, same original data as holotype except for 30.V.2021 • 3♂, same original data as holotype except for 27.V.2025; DNA voucher no. LGE-G1040 • 4 larvae ex *Arachniodes sporadosora*; same original data as holotype except for 16.VI.2025; DNA voucher nos LGE-G1020, -G1022, -G1039, -G1062.

###### Diagnosis.

This new species can be distinguished from its two congeners *P. cuneiformis* (Guan & Li, 2016) and *P. fushana* (Shen & Hsu, 2024) by the shape of the cucullus and aedeagus. In *P. astathmeta*, the cucullus is gradually narrowed and rounded apically; in *P. cuneiformis* it is rectangular, and in *P. fushana* the apex bears a pointed projection. The aedeagus of *P. astathmeta* is much narrower and longer than the latter two species. The female genitalia of *P. astathmeta* have two signa as in *P. cuneiformis*, but the signum near the ductus bursae is smaller and rounded (rather than triangular), and the median signum is narrower than that of *P. cuneiformis*.

###### Description.

**Adult. *Head***: Vertex, frons, and occiput white, sometimes slightly tinged with pale ochre. Labial palpus white, outer margin of segment II rarely mixed with grey. Antenna with scape white; flagellum ochreous white, with short fine ciliation in male.

***Thorax***: Dorsum of thorax and tegula white, sometimes tinged with pale ochre. Legs white, tarsi strongly mixed with light ochre; fore-femur, -tibia and -tarsus black along dorsal margin, this dark coloration sometimes very weak on fore-femur; mid-tibia with a black spot at apex of outer surface and with white bristles dorsally; hind-tibia with whorls of black bristles at apex and with white bristles dorsally, slightly mixed with black beyond middle; hind-tarsus with each tarsomere tipped with dark grey on outer surface.

***Wings*** (Figs 1A, 1B, 2): Forewing span 8.0–10.0 mm (*n* = 31; holotype = 8.5 mm). Forewing ground color white, sometimes sprinkled with grey; costa black in 1/4 wing length, often with a large, indistinct pale ochre patch at middle, this patch sometimes obliquely extended toward dorsal spot; dorsum usually with an elliptical dark grey spot beyond middle, sometimes reduced to a narrow streak along fold; apex tinged with pale ochre; fringe scales greyish white. Hindwing white to grey; fringe scales white to greyish white. Venation: Forewing with 12 veins: Sc reaching basal 1/3 of costa; R1 and R2 separate but both arising from near upper angle of discal cell; R2 parallel to R3–5; R3 short-stalked with long-stalked R4+5; M1 weak; M2 and M3 separate and approximately parallel; CuA1 arising from near lower angle of discal cell; CuA2 absent; 1A+2A unbranched. Hindwing with 10 veins: discal cell open; Rs weak and nearly reaching apex; CuA1 and CuA2 parallel; CuP and 1A+2A weak.

***Abdominal segments*** (Fig. 3A, B): Terga with a row of spines on segments II–VII in male, II–VI in female.

***Male genitalia*** (Fig. 4A): Uncus narrow, tapering toward apex, with long lateral setae from base to near apex; apex blunt, slightly curved downward. Gnathos as long as uncus, elongate-triangular, apex rounded. Tegumen simple. Valva narrowest at base, curved from middle; costa strongly convex near base, with long sparse setae from ~ 1/3 length; cucullus foot-shaped (pediform), covered with dense setae on inner surface, narrowed apically; sacculus nearly straight, with sparse setae ventrally. Vinculum simple, slightly broadened toward saccus; saccus large, tongue-shaped. Juxta trapezoidal. Anellar lobe developed, narrow, with long sparse setae. Aedeagus ~ 3.5× as long as uncus, rod-shaped, apical 1/3 constricted and rugose, apex rounded; cornutus absent.

***Female genitalia*** (Fig. 5A): Papillae anales weakly sclerotized, covered with setae of various lengths. Intersegmental membrane between papillae anales and segment VIII slightly shorter than papillae anales. Tergum VIII somewhat weakly sclerotized, trapezoidal, with long sparse setae along posterior margin. Sternum VIII forming a shield-shaped plate, slightly concave posteriorly, gradually narrowed anteriorly, with long sparse setae along near posterior margin. Apophyses anteriores as long as apophyses posteriores. Ostium bursae large, opening at posterior margin of sternum VII. Antrum broad, cup-shaped, with longitudinal furrows. Ductus bursae shorter than antrum, slightly broadened beyond middle. Corpus bursae elliptical, twice as long as antrum + ductus bursae, with numerous minute spines near entrance of corpus bursae. Two signa present; one near the spined area of corpus bursae, round and deeply emarginated anteriorly; the other situated before middle of corpus bursae, forming a transverse band, broadly convex medially, narrowed and strongly curved laterally toward the former signum. Bulla arising from posterior margin of corpus bursae, stout. Ductus seminalis long, with minute spines apically.

###### Host plant.

Dryopteridaceae: *Arachniodes sporadosora* (Kunze) Nakaike.

###### Biology.

(Fig. 6) Larvae make silky galleries on the underside of fern fronds. Adults fly in May, and larvae are found only in June, suggesting that the species is likely univoltine.

###### Distribution.

(Fig. 7) Korea (Jeju-do).

###### Etymology.

The species name is derived from the Greek *astathmetos* (unstable), referring to the indistinct forewing markings.

###### Remarks.

*Pediformis* is a small genus currently comprising only two species, *P. cuneiformis* and *P. fushana* (Guan and Li 2016; Shen et al. 2024), and *P. astathmeta* is the third taxon described within the genus. Based on a host plant survey conducted on Jeju Island, *P. astathmeta* shares its host plant with *Cuprina fuscella* in the same habitat. However, larvae of *P. astathmeta* were found only on *Arachniodes sporadosora* whereas *C. fuscella* feeds not only on *A. sporadosora* but also on three other fern species of Dryopteridaceae.

#### Genus *Calicotis* Meyrick, 1889

##### Calicotis atricosta sp. nov.

Taxon classificationAnimaliaLepidopteraStathmopodidae

2D86E8F6-AE13-53E3-9249-3FA4C40ED071

https://zoobank.org/02F650B6-44A2-4840-B142-8F3878DA16A2

Figs 1C, 1D, 3C, 4B, 7

###### Korean name.

Geom-eun-tti-hol-ssi-kkok-ji-na-bang.

###### Type material.

***Holotype***: Korea • ♂; Jeju-do, Seogwipo-si, Topyeong-dong; 27.VIII.2021; Ga-Eun Lee leg.; genitalia slide no. LGE22020 (JNU); DNA voucher no. LGE-G901. ***Paratype***: Korea • 1♂; same original data as holotype; genitalia slide no. LGE22019 (NIBR); DNA voucher no. LGE-G549.

###### Additional species examined.

*Calicotis latebrifica*: Korea • 7♂10♀; Jeju-si, Aewol-eup, Gwakji-ri; 28.IV–15.V.2021; Ga-Eun Lee leg.; genitalia slide nos LGE21025m, LGE21030f; DNA voucher no. LGE-G552 • 3♂8♀; Aewol-eup, Aewol-ri; 8,10.V.2021; Ga-Eun Lee leg. • 1♀; Jeju-si, Hangyeong-myeon, Chengsu-ri; 2.VI.2021; Ga-Eun Lee leg.; genitalia slide no. LGE22086f; DNA voucher no. LGE- G899 • 1♀; Seogwipo-si, Namwon-eup, Sumang-ri; 21.VI.2023; Ga-Eun Lee leg.; genitalia slide no. LGE24021f; DNA voucher no. LGE-G900 (all deposited in JNU) • 1 larva ex *Cyrtomium falcatum* var. *devexiscapulae*; Seogwipo-si, Daejeong-eup; 7.VI.2025; Ga-Eun Lee leg.; DNA voucher no. LGE-G1006 • 1 larva ex *Cyrtomium falcatum* var. *devexiscapulae*; Jeju-si, Yeon-dong; 10.VI.2025; Ga-Eun Lee leg.; DNA voucher no. LGE-G1015 • 1 larva ex *Arachniodes aristata*; Seogwipo-si, Andeok-myeon, Seogwang-ri; 22.VI.2025; Ga-Eun Lee leg.; DNA voucher no. LGE-G1038.

###### Diagnosis.

This new species can be readily distinguished by the whitish forewing with a black basal costa and a broad black antemedian fascia. While many congeneric species have a dark blotch or streak beyond the middle of the wing, no markings are present in the distal part of the forewing in this species. In the male genitalia, the oval cucullus and anellar lobe, and the rounded juxta are diagnostic.

###### Description.

**Adult. *Head***: Vertex and occiput mixed creamy white and black, frons creamy white. Labial palpus creamy white, outer margin of segment II tinged with dark grey. Antenna with scape creamy white, posterior margin of scape black; flagellum cream, with short fine ciliation in male.

***Thorax***: Dorsum of thorax and tegula creamy white, anterior margin of tegula black. Legs creamy white; fore-femur, -tibia and -tarsus black along dorsal margin; mid-tibia tinged with black near base and apex of outer surface, with white bristles dorsally; hind-tibia with white bristles dorsally, slightly mixed with grey beyond middle, apex with whorls of ochreous white bristles; hind-tarsomeres I–IV with whorls of ochreous white bristles at apexes.

***Wings*** (Fig. 1C, D): Forewing span 7.5 mm (*n* = 2). Forewing ground color creamy white, slightly mixed with grey toward apex; costa black in 1/4 wing length, this dark shadow expanded as a basal patch and nearly reaching fold; beyond this, a broad, inwardly oblique black antemedian fascia running from costa to dorsum, broadest near costa and narrowest on fold; fringe scales greyish white. Hindwing dark grey except base white; fringe scales greyish white.

***Abdominal segments*** (Fig. 3C): Terga with a row of spines on segments II–VII in male.

***Male genitalia*** (Fig. 4B): Uncus stout, gradually tapering, with long lateral setae from base to near apex; apex blunt, slightly curved downward. Gnathos slightly shorter than uncus, tongue-shaped, apex rounded. Tegumen simple. Valva somewhat narrowed toward apex; costa slightly convex near base, with long sparse setae at ~ 1/3 length; cucullus oval, covered with dense setae on inner surface; sacculus nearly straight from base to near apex, rounded apically, with long sparse setae ventrally. Vinculum narrow and elongate, leading to rounded saccus. Juxta rounded. Anellar lobe developed, oval, with short setae. Aedeagus ~ 3× as long as uncus, stout, distal half tapering toward apex; a narrow apical spatulate projection present, ~ 1/3 length of aedeagus, with apex rounded; cornutus absent.

***Female genitalia***. Unknown.

###### Host plant and biology.

Unknown.

###### Distribution.

(Fig. 7) Korea (Jeju-do).

###### Etymology.

The species name is derived from the Latin *ater* (black) and *costa* (costa), referring to the black basal costa of the forewing.

###### Remarks.

Due to the limited number of available specimens, no wing venation slide was prepared for the new species in order to avoid destructive sampling of the type material.

#### Genus *Cuprina* Sinev, 1988

##### Cuprina porphyrantha

Taxon classificationAnimaliaLepidopteraStathmopodidae

(Meyrick, 1913)

0BC62FA9-D397-5309-B52F-0A7E4072BA49

Figs 1E, 3D, 4C, 7

Stathmopoda porphyrantha Meyrick, 1913: 90. TL: Ceylon (Sri Lanka). TD: NHMUK.
Cuprina porphyrantha (Meyrick): Shen and Hsu 2020: 118; Kobayashi and Terada 2022: 112; Shen et al. 2024: 10.
Cuprina flaviscapella Sinev, 1988: 126; Terada 2016: 150. Synonymized by Sinev (2015: 24).

###### Korean name.

Gwan-jung-ae-gi-kkok-ji-na-bang.

###### Material examined.

Korea • 1♂; Jeju-do, Seogwipo-si, Topyeong-dong; 27.VIII.2021, Ga-Eun Lee leg.; genitalia slide no. LGE22033 (NIBR); DNA voucher no. LGE-G548 • 1 larva ex *Arachniodes aristata*; Seogwipo-si, Andeok-myeon, Seogwang-ri; 22.VI.2025; Ga-Eun Lee leg.; DNA voucher no. LGE-G1023.

###### Additional species examined.

*Cuprina fuscella*: Korea • 2♂; Jeju-do, Seogwipo-si, Topyeong-dong; 22.IV,23.V.2021; Ga-Eun Lee leg.; genitalia slide no. LGE21029 (JNU); DNA voucher no. LGE-G738 • 4 larvae ex *Arachniodes sporadosora*, *Cyrtomium falcatum* var. *devexiscapulae*, *Dryopteris uniformis* and *Polystichum polyblepharum*; Jeju-do, Seogwipo-si, Topyeong-dong; 30.V.–19.VI.2025; DNA voucher nos LGE-G1000, -G1001, -G1014, -G1021 • 1 larva ex *Dryopteris uniformis*; Jeju-do, Jeju-si, Ara-dong; 6.VI.2025; Ga-Eun Lee leg.; DNA voucher no. LGE-G1005 • 1 larva ex *Dryopteris uniformis*; Jeju-do, Jeju-si, Yeon-dong; 10.VI.2025; Ga-Eun Lee leg.; DNA voucher no. LGE-G1016 • 3 larvae ex *Dryopteris uniformis* and *Polystichum polyblepharum*; Jeju-do, Jeju-si, Jocheon-eup, Gyorae-ri; 11.VI.2025; Ga-Eun Lee leg.; DNA voucher nos LGE-G1017, -G1018, -G1019.

###### Diagnosis.

Forewing span 6.9 mm (*n* = 1). This species closely resembles *C. fuscella* in both external appearance and genitalia. It is difficult to distinguish the two species based on superficial appearance, as both have dark forewings without distinct markings. However, antennal coloration provides a useful diagnostic character. The scape of *C. porphyrantha* is ochreous yellow dorsally (Fig. 1E); in *C. fuscella* it is fuscous to dark grey. Also, the apical portion of the antennae is more broadly pale in *C. porphyrantha*, although this character may sometimes be indistinct. In the male genitalia (Fig. 4C), the basal sclerotized structure of the aedeagus is larger in *C. porphyrantha* than in *C. fuscella*. The female genitalia of the two species were not examined in this study; however, according to Terada (2016), the signum of *C. porphyrantha* is smaller.

###### Host plants.

Dryopteridaceae: *Dryopteris crassirhizoma* Nakai (Terada 2016), *D. hondoensis* Koidz. (Kobayashi and Terada 2022), *Dryopteris* sp. (Sawamura et al. 2009; *C. porphyrantha* was identified as Stathmopodinae sp.), *Arachniodes aristata* (G.Forst.) Tindale (this study).

###### Biology.

Larvae are found in silky galleries on the underside of fern fronds. Adults fly between May and August in Japan (Terada 2016). In Korea, the larvae are found in June and adults in August.

###### Distribution.

(Fig. 7) Korea (new record), Japan, Russia, Sri Lanka, Taiwan.

###### Remarks.

Detailed descriptions of the adults and genitalia are provided in Terada (2016). The larvae of *C. porphyrantha* are specialists on Dryopteridaceae whereas the closely related species *C. fuscella* feeds not only on Dryopteridaceae but also on two other families Athyriaceae and Onocleaceae (Sinev 1988; Sawamura et al. 2009; Terada 2016). Shen and Hsu (2020) listed Theyriaceae and Woodsiaceae as host plants of *C. fuscella*, but these were included in error (Shen pers. comm. 6 Jun 2025).

#### Genus *Petala* Shen, Terada & Hsu, 2024

##### Petala aedificatrix

Taxon classificationAnimaliaLepidopteraStathmopodidae

(Terada, 2016)

67BB030A-4308-5503-83D2-8158AD4F18E5

Figs 1F, 3E, 4D, 7

Pachyrhabda aedificatrix Terada, 2016: 111. TL: Honshu, Japan. TD: OMU.
Petala aedificatrix : Shen et al. 2024: 20.

###### Korean name.

Nam-bang-go-sa-ri-kkok-ji-na-bang.

###### Material examined.

Korea • 1♂; Jeju-do, Jeju-si, Jocheon-eup, Gyorae-ri; 6.VI.2023; Ga-Eun Lee leg.; genitalia slide no. LGE24019 (NIBR); DNA voucher no. LGE-G897.

###### Diagnosis.

Forewing span 10.0 mm (*n* = 1). This species is superficially similar to *P. margaritacea* (Terada 2016), but the forewing span is larger and the markings are somewhat indistinct in *P. aedificatrix* (Fig. 1F). In addition, *P. aedificatrix* has black bristles at the apex of the hind-tibia; those of *P. margaritacea* are white to pale grey. In the male genitalia of *P. aedificatrix* (Fig. 4D), the cucullus is slightly curved outward apically and the sacculus is indistinct; in *P. margaritacea*, the cucullus is not curved apically and the sacculus has a rounded apex. The aedeagus of *P. aedificatrix* is distinctive in having a needle-like cornutus with a sharply pointed apex, situated in the distal half of the aedeagus.

###### Host plants.

Dryopteridaceae: *Arachniodes amabilis* (Blume) Tindale, *Cyrtomium fortune* J. Sm., *Dryopteris erythrosora* (D.C.Eaton) Kuntze, *D. hondoensis* Koidz., *D. nipponensis* Koidz., *D. pacifica* (Nakai) Tagawa; Athyriaceae: *Cornopteris decurrenti-alata* (Hook.) Nakai (Terada 2016).

###### Biology.

Larvae spin a forking silky gallery on the underside of fern fronds. Adults have been collected in May and June in Japan (Terada 2016).

###### Distribution.

(Fig. 7) Korea (new record), Japan.

###### Remarks.

Detailed descriptions of the adult and genitalia are provided in Terada (2016). The genus *Petala* currently includes three species, *P. aedificatrix*, *P. margaritacea*, and *P. shida*.

##### Petala margaritacea

Taxon classificationAnimaliaLepidopteraStathmopodidae

(Terada, 2016)

40806315-67FC-5979-B80B-1A47236F0EF3

Figs 1G, 1H, 3F, 4E, 5B, 7

Pachyrhabda margaritacea Terada, 2016: 115. TL: Kyushu, Japan. TD: OMU.
Petala margaritacea : Shen et al. 2024: 20.

###### Korean name.

Byeol-go-sa-ri-kkok-ji-na-bang.

###### Material examined.

Korea • 6♂4♀; Jeju-do, Jeju-si, Yeon-dong; 24.VIII.2020–24.VI.2024; Ga-Eun Lee leg.; genitalia slide nos LGE20014m, LGE20015f, LGE24022m; DNA voucher nos LGE-G551, LGE-G894 • 1♂; Jeju-do, Jeju-si, Hallim-eup, Gwideok-ri; 12.VII.2022; Ga-Eun Lee leg.; genitalia slide no. LGE20018; DNA voucher no. LGE-G896 • 1♀; Jeju-do, Jeju-si, Hallim-eup, Dongmyeong-ri; 22.VI.2023; Ga-Eun Lee leg.; genitalia slide no. LGE24020; DNA voucher no. LGE-G898 (all deposited in JNU except for one male in NIBR).

###### Diagnosis.

Forewing span 6.5–7.5 mm (*n* = 12). *Petala margaritacea* is closely related to *P. aedificatrix* and the differential diagnosis between the two species is stated under the latter.

###### Host plant.

Thelypteridaceae: *Christella acuminata* (Houtt.) Holttum (Terada 2016).

###### Biology.

Larvae make silky galleries on the underside of fern fronds as in *P. aedificatrix*. Adults have been collected from April to October in Japan (Terada 2016).

###### Distribution.

(Fig. 7) Korea (new record), Japan.

###### Remarks.

Detailed descriptions of the adult and genitalia are provided in Terada (2016). The host plant has been referred to as *Thelypteris acuminata* (Houtt.) Morton in Japan (Terada 2016) and as *Cyclosorus acuminatus* (Houtt.) Nakai in Korea (National Institute of Biological Resources 2025). Here, we adopt *Christella acuminata* (Houtt.) Holttum following recent global taxonomic treatments (WFO 2026).

## Phylogenetic results and discussion

**Figure 8.**
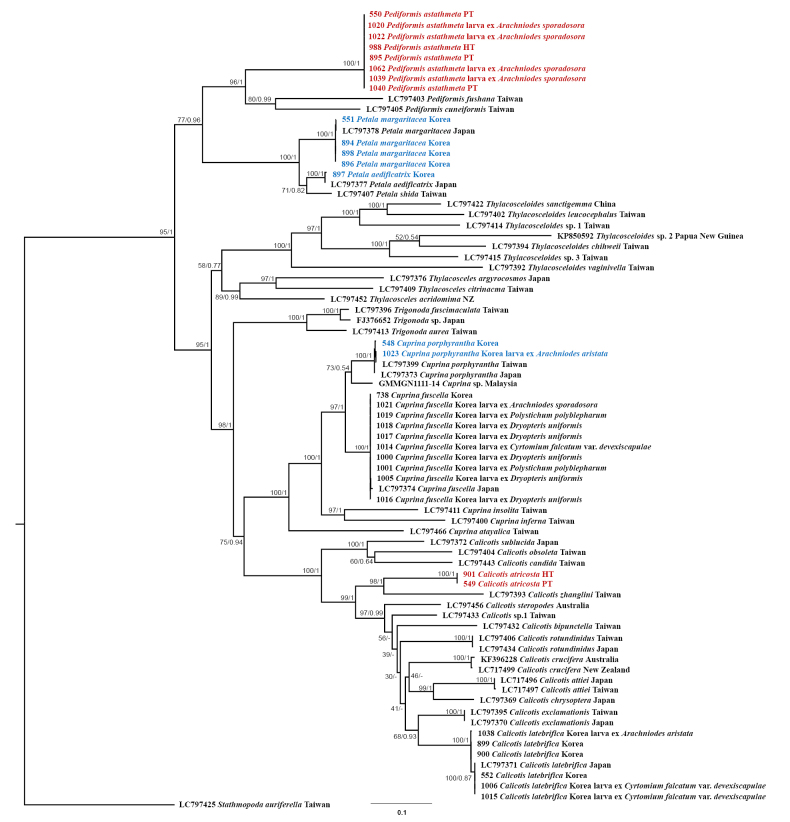
Maximum likelihood tree of Cuprininae inferred using IQ-TREE. Ultrafast Bootstrap (UFBoot) values and Bayesian posterior probabilities (PP) are indicated at each node. Nodes not recovered in the Bayesian analysis are indicated by dashes (–). Newly described species are shown in red, and newly recorded species in blue.

The ML and BI analyses based on the concatenated five-gene dataset yielded largely congruent topologies, and all seven taxa examined here were consistently assigned to their respective genera (Fig. 8). Topological differences between ML and BI analyses were limited to the deepest relationships within *Calicotis*, which were collapsed in the BI tree; the placement of all Korean taxa remained identical between analyses. In *Pediformis*, the new species *P. astathmeta* sp. nov. was nested within a strongly supported *Pediformis* clade (UFBoot = 96, PP = 1), confirming its generic placement and recovering it as sister to *P. fushana* + *P. cuneiformis*. Similarly, *Calicotis atricosta* sp. nov. was placed within the *Calicotis* clade (UFBoot = 100, PP = 1) and was sister to *C. zhanglini* with strong support (UFBoot = 98, PP = 1). The three newly recorded species (*Petala aedificatrix*, *P. margaritacea*, and *Cuprina porphyrantha*) were grouped with previously identified conspecific sequences from other regions, supporting their species-level identification. Despite incomplete nuclear gene coverage for several specimens, the placement of the Korean taxa remained consistent across ML and BI analyses and congruent with morphological evidence.

At the intergeneric level, relationships among the sampled genera largely matched those reported by Shen et al. (2024), except for the relationships among *Calicotis*, *Cuprina*, and *Trigonoda*, which showed incongruence between ML and BI analyses in that study. In the present study, both analyses supported *Calicotis* + *Cuprina* as sister taxa (UFBoot = 75, PP = 0.94), with *Trigonoda* forming the sister lineage to this clade. This topology is identical to that recovered in the ML analysis of Shen et al. (2024), whereas their BI analysis supported an alternative relationship in which *Calicotis* was placed as sister to *Cuprina* + *Trigonoda* (PP = 0.86).

The agreement between ML and BI results suggests that this topology may be robust across analytical methods under the current dataset; however, support for the deeper intergeneric relationships remains moderate. Although posterior probabilities were relatively high, bootstrap support was moderate, indicating that these relationships should still be interpreted cautiously. Shen et al. (2024) included representatives of multiple stathmopodid subfamilies and reconstructed relationships within a broader family-level framework, whereas the present dataset was restricted to Cuprininae and focused primarily on verifying the placement of the Korean taxa. Differences in taxon sampling and dataset composition may therefore explain the discrepancies observed between studies, and broader sampling will be necessary to clarify their evolutionary relationships.

Overall, the phylogenetic analyses based on the multilocus dataset provide consistent support for the taxonomic conclusions of this study. The generic placement of the two new species and the identification of three newly recorded taxa are well supported. Although deeper intergeneric relationships remain subject to further investigation, the present study provides additional molecular data for Cuprininae and contributes to a more robust framework for understanding the diversity of the group in Korea.

## Supplementary Material

XML Treatment for Pediformis astathmeta

XML Treatment for Calicotis atricosta

XML Treatment for Cuprina porphyrantha

XML Treatment for Petala aedificatrix

XML Treatment for Petala margaritacea

## Additional information

### Artificial Intelligence (AI) use

The authors accept full responsibility for the content of the manuscript, including the disclosure of any use of AI.

Regarding the use of AI in the preparation of this manuscript, the authors declare the following: ChatGPT.

**Used for**: English language editing and proofreading.

AI tools were used solely for English language editing and proofreading. All scientific content, including data analysis, interpretation, and conclusions, was developed exclusively by the authors.

### Author ORCIDs

G.-E. Lee https://orcid.org/0000-0002-5774-5850

T. Terada https://orcid.org/0009-0006-5410-6902

### Data availability

All of the data that support the findings of this study are available in the main text.
